# Economic evaluation protocol for the PRevention Of sudden cardiac death aFter myocardial Infarction by Defibrillator implantation: the PROFID EHRA trial

**DOI:** 10.1136/bmjopen-2024-097495

**Published:** 2026-01-08

**Authors:** Yirui Qian, Carlos Rojas Roque, Beth Woods, Cynthia P Iglesias Urrutia, Vijay S Gc, Maria Gur Arie, Daniela Fischer, Nikolaos Dagres, Gerhard Hindricks, Andrea Manca

**Affiliations:** 1 Centre for Health Economics, University of York, York, England, UK; 2 Department of Health Sciences, University of York, York, England, UK; 3 German Heart Center of the Charité, Berlin, Germany

**Keywords:** Defibrillators, Death, Sudden, Cardiac, Randomized Controlled Trial, HEALTH ECONOMICS

## Abstract

**Introduction:**

The implantable cardioverter defibrillator (ICD) is a cardiac device recommended for use to prevent the occurrence of sudden cardiac death (SCD) in post-myocardial infarction (MI) patients with reduced left ventricular ejection fraction (LVEF). The evidence informing this guidance comes from landmark trials that are now more than 20 years old. The risk-benefit profile of ICD for the contemporary target population may have changed substantially since then, which raises the question of whether there is evidence for sparing patients a procedure associated with potentially severe complications and high healthcare costs. A main part of the PRevention Of sudden cardiac death aFter myocardial Infarction by Defibrillator implantation (PROFID) project is the PROFID EHRA trial, which is supported by the European Heart Rhythm Association. PROFID EHRA is a European Union-funded, prospective, randomised, multi-centre, non-inferiority study designed to compare optimal medical therapy (OMT) alone to ICD with OMT, for post-MI patients with reduced LVEF. The study also describes economic evaluation methods to quantify the cost and health implications of using OMT alone in place of ICD implantation plus OMT in this group of patients.

**Methods and analysis:**

The economic evaluation has been designed to conduct a pre-trial cost-effectiveness analysis (CEA) prior to the availability of trial data, followed by a within-trial cost-consequences analysis (CCA) and a long-term post-trial CEA, conducted from the National Health Service and Personal Social Service perspective in England. The pre-trial CEA uses simulation modelling informed by available evidence to assess the lifetime costs and quality-adjusted life years of OMT alone and ICD+OMT in post-MI patients with reduced LVEF at risk of SCD, as defined in the PROFID EHRA trial. The within-trial CCA is intended to summarise the health-related quality of life (HRQoL), healthcare resource use and associated costs observed during the PROFID EHRA trial follow-up period. The post-trial CEA updates the pre-trial model by incorporating contemporary evidence about the HRQoL and costs observed during the trial and the occurrence of those events and outcomes accruing during the trial follow-up period and projecting them into the expected lifetime of the patients. Sensitivity analyses are performed to assess the robustness of the CEA results with respect to both model assumptions and uncertainty in the value of the model input parameters. Finally, a value of information analysis will identify the key drivers of uncertainty surrounding the model conclusions regarding the optimal treatment strategy, establishing if further research may be required.

**Ethics and dissemination:**

The PROFID EHRA trial, under legal sponsorship of Charité—Universitätsmedizin Berlin, Germany, received its first ethics approval by the Medicine Research Ethics Committee of the La Paz University Hospital in Madrid, Spain (reference number LHS-2019-0209). Before including patients, for all participating study centres, the required local, central and/or national ethical approval has to be obtained. As of the date 13 November 2025, at least one participating study centre in the following countries has received ethical approvals from relevant ethics committees: Austria, Belgium, Czech Republic, Denmark, France, Germany, Great Britain, Hungary, Israel, the Netherlands, Poland and Spain. Results will be shared with the general public through various media channels and additionally with healthcare professionals and the scientific community through scientific meetings, conferences and publications.

**Trial registration number:**

NCT05665608.

**Strengths and limitations of this study**

- This protocol describes the design of an economic evaluation study to assess the contemporary health benefits and costs of routine prophylactic implantable cardioverter defibrillator (ICD) for the PROFID EHRA trial.
- The proposed economic evaluation methods include a pre-trial cost-effectiveness analysis (CEA) prior to the completion of the PROFID EHRA trial, a within-trial cost-consequences analysis (CCA) and a post-trial CEA that incorporates data from the PROFID EHRA trial.
- The pre-trial CEA constructs a simulation model using available evidence to inform the prediction of expected health outcomes and costs associated with the treatment strategies investigated in PROFID EHRA, with the relative treatment effect of ICD implantation on all-cause mortality informed by structured expert elicitation methods.
- The post-trial CEA updates parameter inputs of the pre-trial simulation model with health-related quality of life (HRQoL), fatal and non-fatal events and costs derived from the analysis of the data collected in the PROFID EHRA trial.
- A value of information analysis will assess the expected gain from reducing uncertainty through the collection of more information on key parameters and determine whether it is worth investing resources to obtain more information on these by funding further research.

## Background

 Sudden cardiac death (SCD) is a major public health problem, accounting for around 50% of cardiac fatalities and 10%–20% of all deaths in Europe.^1^,^5^ It is reported that the overall incidence of SCD for patients who have survived a myocardial infarction (MI) ranges from 2% to 4% per year, with significantly increased risk within the first few months post MI.^6^ Coronary artery disease is believed to be responsible for about 3/4 of SCD cases.^5^ A severely reduced left ventricular ejection fraction (LVEF) serves as a general indicator of impaired heart function after MI and has been used to signal heightened risk for SCD.^5^ The implantable cardioverter defibrillator (ICD) is a cardiac device designed to identify and halt ventricular tachyarrhythmias using methods such as rapid ventricular pacing and shocks, and ultimately prevent SCD among post-MI patients with reduced LVEF.

Two landmark randomised controlled trials (RCTs) were conducted between the end of the 1990s and early 2000s, that is, the Multicenter Automatic Defibrillator Implantation Trial (MADIT-II)^7^ and the Sudden Cardiac Death in Heart Failure Trial (SCD-HeFT).^8^ These trials compared optimal medical therapy (OMT) alone to ICD with OMT, in patients with severely impaired LVEF, either after MI or due to other causes of heart failure. Both studies reported significantly higher survival rates for patients receiving ICD alongside OMT, compared with those receiving OMT alone, which led to international guidelines recommending routine implantation of ICDs in patients with a prior MI and severely reduced LVEF.^5 9^ The question is whether current evidence continues to provide support for this guidance, or whether there is now a case for sparing patients a procedure associated with potentially severe complications and high healthcare costs.

Several studies show that the risk-benefit of ICD implantation for primary prevention of SCD in patients with severely reduced LVEF has substantially changed in the last two decades.^10 11^ First, both SCD and all-cause mortality have decreased over the last decades as a result of the advances in medical treatment for heart failure.^12^ At the same time, the appropriate shock delivered by prophylactic defibrillators such as ICD has decreased from nearly 17% to 1%–3% per year.^13^ Lastly, multiple studies demonstrate that the complication rate of ICD therapy remains a relevant concern.^14^,^18^ Thus, there is an urgent need for a novel, well-designed, adequately powered RCT to evaluate the effectiveness of the ICD therapy under contemporary OMT.^19 20^

The PRevention Of sudden cardiac death aFter myocardial Infarction by Defibrillator implantation European Heart Rhythm Association (PROFID EHRA) trial aims to reassess the potential benefits and harms of routine prophylactic ICD implantation in post-MI patients with reduced LVEF ≤35% alongside contemporary OMT for primary prevention of SCD in an RCT setting.^21^ The trial is part of the PROFID project.^22^ The project includes an economic evaluation to quantify the cost and health implications of using OMT alone in place of ICD implantation plus OMT in this group of patients. This trial is currently ongoing.

This paper describes the protocol of the economic evaluation study designed to identify, measure and value the healthcare resource use and health benefits associated with the treatment strategies compared in the trial using an evidence-based iterative approach, updating the economic analysis as new evidence becomes available. First, we structure and populate the PROFID EHRA health economics model(s) using existing sources of evidence, prior to the data and results of the trial becoming available. Next, we conduct a within-trial cost-consequence analysis (CCA) and a post-trial cost-effectiveness analysis (CEA), with the latter carried out after updating the model input parameters with data from the PROFID EHRA trial where applicable. The results of the economic evaluation are expected to contribute to informing whether the implantation of an ICD for primary prevention of SCD in post-MI patients with reduced LVEF, along with OMT, is a cost-effective strategy.

## Methods

### PROFID EHRA trial design

PROFID EHRA is a non-commercial, investigator-driven, prospective, parallel-group, randomised, open-label, blinded outcome assessment, multi-centre, non-inferiority trial with 1:1 allocation between two treatment groups. The trial is event-driven to test whether OMT alone is not inferior to OMT with ICD implantation in post-MI patients with LVEF ≤35% (referred to here as ‘reduced LVEF’) with respect to all-cause mortality within 2.5 years of observation post index date. Recruitment of patients for the study started in November 2023, with the aim to enrol 3 595 patients in 180 participating study sites in Europe and in Israel. The total sample size of 3 595 patients was determined by the responsible PROFID EHRA statistician based on formal sample size calculations.^21^ As of 20 October 2025, 256 of the planned 3 595 patients had been enrolled in 83 of 180 planned participating sites.^21^ Secondary prevention ICD implantations in patients initially randomised to the OMT arm are considered as a potential ‘real world’ consequence of the initial strategy rather than crossovers,^21^ which is consistent with the trial intention-to-treat analysis. Study details of the PROFID EHRA trial design are published in the study protocol of the trial^21^ and on the clinical trial registry website.^23^

### Decision problem

The decision problem of this economic evaluation is whether OMT alone is a cost-effective strategy compared with ICD+OMT for post-MI patients with reduced LVEF, from the perspective of the National Health Service (NHS) and Personal Social Service (PSS) in England. The economic evaluation study described in this protocol estimates the relevant outcomes—such as overall survival, health-related quality of life (HRQoL) and costs associated with resources used—for the health states in the model, to assess whether the contemporary evidence supports continued ICD use with OMT, or whether OMT alone is a viable and cost-effective alternative.

### Economic evaluation methods

The economic evaluation includes a pre-trial CEA, a within-trial CCA and a post-trial CEA. The pre-trial economic analysis structures and populates the simulation model using external data sources to estimate the long-term costs and quality-adjusted life years (QALYs) of OMT alone versus ICD use with OMT. Based on longitudinal individual-level data collected during the PROFID EHRA trial follow-up, the within-trial CCA summarises patients’ HRQoL and costs observed during the study follow-up period. The post-trial CEA revises the simulation using updated model input parameters obtained, wherever possible, from the PROFID EHRA trial data.

**Figure 1:**
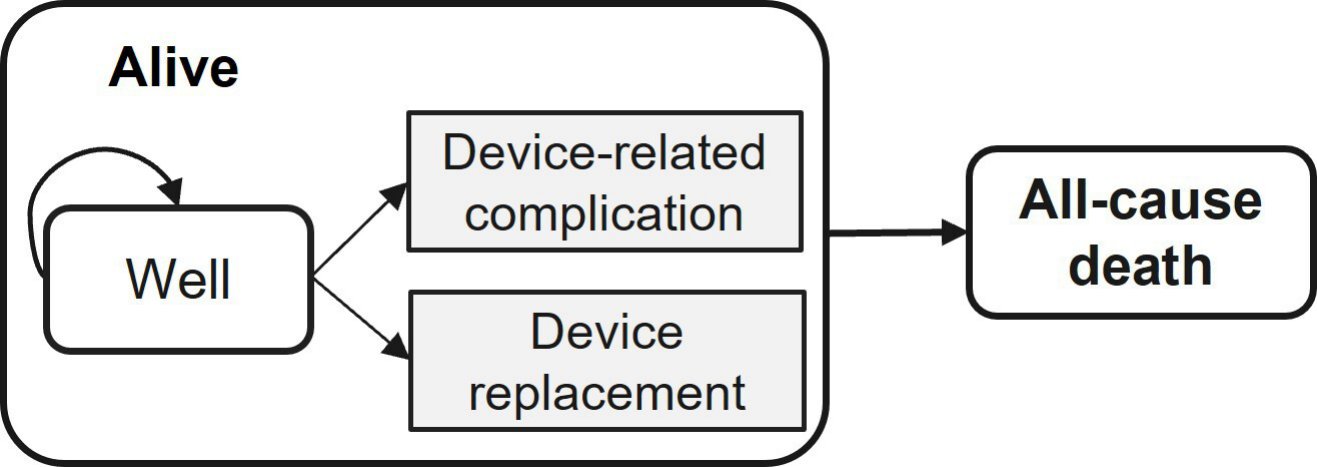
The rounded rectangles represent two health states (ie, Alive/Well and All-cause death), rectangles represent two transient events (ie, Device-related complication and Device replacement), arrows point the transition from Alive to All-cause death in one direction, and the circular arrow represents remaining in the same state.

Even when individual patient-level data are available from the PROFID EHRA trial, it is realistic to expect that resource use and health consequences associated with the treatment choice may extend beyond the study follow-up. The clinical event of interest (eg, mortality) may not yet have occurred for every patient in the study by the end of the follow-up period. This means that the estimation of the average overall survival and the overall rate of occurrence of clinical events—such as complications and device replacement—will have a certain range of uncertainty. Similarly, the total costs and HRQoL trajectories for participants who are still alive at the end of the trial will not be fully observed. Ignoring censoring in these outcomes may bias the estimation of the mean outcome and cost estimates.^24^ Methodological guidance in this area recommends that these outcomes should be projected through the life course of the patient(s), to ensure that all the effects of the interventions on the total costs and QALYs have been reflected by that time.^25^ Hence, a two-state model (Figure 1) is developed to estimate the long-term average costs and QALYs for the two treatment groups in the PROFID EHRA trial. Its structure is informed by a review of the published models that have been used previously to evaluate the cost-effectiveness of ICD implantation for the prevention of SCD.^26^ The face validity of the conceptual model has been tested by presenting it to clinical experts from the PROFID EHRA team.

Patients enter the model in the ‘Alive’ state and remain there until (all-cause) death occurs, which is represented by the arrow pointing from the ‘Alive’ state to the ‘All-cause death’ state. The risk of mortality is assumed to vary between individuals as a function of their baseline characteristics and exposure to treatment. The estimation of this risk is described in the Analysis section. While in the ‘Alive’ state, patients may be event-free (represented by the sub-state ‘Well’) or experience transient events. Transient events are health occurrences that may lead to increased costs and/or reduced HRQoL for a duration of time, but that are assumed to be neither permanently disabling in nature nor to structurally modify the risk of all-cause mortality. In the proposed CEA model, patients in the OMT with ICD group can experience transient events such as device-related complications and device replacements.

The CEA models take a lifetime horizon that projects patient outcomes over their entire life course till death. HRQoL and costs are modelled to accrue at discrete time intervals while patients are in the ‘Alive’ state. The total costs and QALYs for each treatment group are obtained by integrating the costs accumulated during the simulation period. Details about the derivation of the input model parameters for these outcomes, as well as the probabilities governing the occurrence of fatal and non-fatal events, for both pre-trial and post-trial CEA, are provided in the Analysis section.

### Quality of life

HRQoL used in the PROFID EHRA trial is measured by the index score of the 5-level EuroQoL 5-dimensions (EQ-5D-5L), a preference-based generic patient-reported outcome comprising five dimensions: mobility, self-care, usual activities, pain/discomfort and anxiety/depression.^27^ Each dimension has five levels of severity, ranging from 1 (no problems) to 5 (extreme problems), the combination of which yields a total number of 3125 possible health states.^28^ Index scores can be calculated from these health states, and we shall follow the relevant methodological guidance at the time of analysis to generate the appropriate values for the EQ-5D index scores from the EQ-5D-5L index scores for use in this economic evaluation study. At present, in England, the guidance for health technology evaluations, provided by the National Institute for Health and Care Excellence (NICE),^29^ recommends generating index scores by mapping the EQ-5D-5L responses onto the EQ-5D-3L value sets using the approach developed by Hernández Alava *et al*.^30^

### Costs

Healthcare resource use measured in the PROFID EHRA trial includes lab tests undertaken, imaging, medication, ICD device and its implantation, complications, and replacement, hospitalisation and clinical follow-up visits. In the absence of patient-level data from PROFID EHRA, we will use a combination of figures from the National Cost Collection for NHS,^31^ clinical expert advice and published evidence to estimate the cost (and relevant measures of uncertainty) associated with the membership of the health states represented in Figure 1. Device cost will be retrieved from the average selling price requested through the Association of British Healthcare Industries.

### Patient and public involvement

Patients and/or the public are not involved in the design, or conduct, or reporting or dissemination plans of this research.

## Analysis

The statistical analyses of the individual patient data from the trial as well as the CEA model will be programmed in R^32^ using RStudio^33^. A discount rate of 3.5% will be applied to both costs and health outcomes, following the current NICE Guidance.^29^

### Long-term model-based pre-trial analysis

The time that patients spend in each of the health states and the transient events they may experience during their lifetime is modelled through a series of risk equations. The risks of transient events will be adapted from published evidence. To estimate transition probabilities for all-cause mortality conditional on patients’ characteristics, we will analyse individual patient-level data from the Swedish Heart Registry (SWHR), with the sample selected to match as closely as possible, depending on data availability, the inclusion/exclusion criteria used in PROFID EHRA. This registry is a combination of several data sources: the Swedish Web-System for Enhancement and Development of Evidence-Based care in Heart Disease Evaluated According to Recommended Therapies (SWEDEHEART) registry including data from the Register of Information and Knowledge About Swedish Heart Intensive Care Admissions (RIKS–HIA), the Swedish Coronary Angiography and Angioplasty Registry, and the Swedish Heart Surgery Registry; the Swedish cardiopulmonary resuscitation registry; and the Swedish Pacemaker and ICD registry.^34^ Data were collected from 2006 to 2017, and the follow-up start was set to 40 days from acute MI hospital admission. Data from the SWHR will be used to estimate the parameters to model the prognosis of the non-ICD patients.^35^ Parametric survival models will be used to estimate and extrapolate the risk of all-cause mortality.

The treatment effect of the ICD implantation on mortality will be estimated using structured expert elicitation, aiming to systematically collect individual judgments and mitigate bias.^36^ Experts will review the evidence from MADIT-II and SCD-HeFT alongside more contemporary information about device therapy and OMT, before formulating their judgement about the contemporary effects of ICD therapy. Synthesis using mathematical aggregation (eg, linear opinion pooling) or behavioural methods (eg, consensus discussions) combines experts’ individual opinions into a collective and quantifiable representation. For this task, we will follow the protocols and methods described in Bojke *et al*.^36^ A series of questions will be used to elicit the size and duration of the treatment effect of ICD implantation compared with OMT on all-cause mortality. This will then be applied to the estimate of baseline risk from the SWHR to parametrise a risk equation for the ICD+OMT arm from which we will estimate survival probabilities.

Patient-level EQ-5D data from the EMMACE register,^37^ a prospective cohort that documents clinical events and HRQoL for post-MI patients in the UK, will be analysed to estimate the mean EQ-5D, together with the relevant measure of sample uncertainty, associated with each of the health states and events being modelled. The costs in the pre-trial CEA will be informed by published evidence.

### Within-trial cost-consequences analysis

Individual-level HRQoL and healthcare resource use data in the PROFID EHRA trial are collected via an electronic case report form which is completed at the baseline, 12-month and 24-month clinical visits.^38^ The CCA will provide information on HRQoL, healthcare resource use and associated costs for the two strategies being compared in the trial during the PROFID EHRA follow-up period. The average resource use consumption, total costs and EQ-5D will be summarised (alongside relevant measures of dispersion) at baseline (EQ-5D only) and at follow-up for the two treatment groups separately. The trial is not powered to detect statistically significant differences in costs and HRQoL—since the sample size calculation was based on overall survival as its primary outcome—and so no formal statistical testing for difference in the health economic outcomes between groups will be carried out in the CCA.^39 40^

### Long-term model-based post-trial analysis

A range of statistical techniques will be applied to derive the parameter inputs to populate the long-term CEA model, which will include transition probabilities from ‘Alive’ to ‘All-cause death’, probabilities or rates of transient event occurrence in ‘Alive/Well’ state and costs and EQ-5D associated with ‘Alive/Well’ state and transient events.

#### Modelling all-cause mortality

Time-to-death from all causes will be analysed using standard parametric survival models where the baseline hazard function is specified using a parametric distribution, such as the exponential, Weibull, Gompertz, log-Normal or Gamma.^41 42^ Predictions from these models will be used to extrapolate the events of interest that are not fully observed during the trial period, to estimate the overall survival associated with each treatment group. Outputs from the survival regression models will be used to derive the transition probabilities from ‘Alive’ to ‘All-cause death’. Model selection will be informed by assessing internal and external validity using long-term mortality evidence from relevant cohorts.^41 43 44^ Flexible parametric survival models (eg, spline models) or the integration of external evidence will also be considered if the hazard function is complex and cannot be adequately reflected using standard models.^45^,^47^

Additionally, the treatment effect of the ICD implantation on mortality will be assumed to be constant for a certain number of years after which it will gradually diminish to zero. This is called the waning of treatment effect, as a conservative method to estimate the long-term treatment effect on overall survival due to the duration of the PROFID EHRA trial observation period.^48^

#### Modelling transient events

Similar to the analysis of all-cause mortality, survival analysis can be conducted to analyse transient events using time-to-device-related complications and time-to-device-replacement endpoints analysed using two separate survival regressions. The probabilities or event rates of transient events will be estimated using evidence from the PROFID EHRA trial, or external sources if this input cannot be accurately informed from trial data. Although transient events may lead to increased costs and reduced EQ-5D, their occurrence is assumed not to be structurally linked to all-cause mortality due to the difficulty of estimating mortality conditional on these relatively infrequent events in a trial of this size.

The following device-related complications will be considered for the analysis: lead failure, tamponade, pneumothorax, dislocation of lead, significant pocket haematoma and infection, as validated by clinical experts from the PROFID EHRA trial.

#### Modelling cost and EQ-5D data

Cost and EQ-5D will be analysed using generalised linear regression models and their extensions, to account for the non-normal distribution of the outcome, the presence of spikes in the distributions (eg, at zero for the costs and at one for the EQ-5D) and the role of baseline predictors. The regression outputs will inform the quantification of the cost-effectiveness model parameters, which will help predict the long-term mean cost and QALYs for the strategies being compared in the PROFID EHRA trial.

The costs and EQ-5D for the ‘Well’ state will be populated within the long-term CEA model using the value of their conditional predicted means from the regression analyses. The costs and EQ-5D associated with the occurrence of transient events will be calculated as cost increments or disutility associated with specific events.

### Prognostic variables

Based on feedback from clinical experts in the PROFID EHRA trial, the regression analyses for overall survival, costs and EQ-5D will consider the following prognostic variables as predictors: age at baseline, gender, race, LVEF, New York Heart Association class, history of atrial fibrillation, renal dysfunction, QRS interval, medically treated diabetes and history of MI.

Depending on the distribution of patients’ recruitment across centres and countries, the feasibility of a multilevel modelling approach to the regression analyses will be considered, to account for the hierarchical structure of individual-level data, which will be naturally nested within centres and countries.^49^ Missing outcome data in the PROFID EHRA trial, particularly in the resource use and EQ-5D, will be assessed and if necessary handled with appropriate strategies,^50^ such as a multiple imputation via chained equations approach.^51^

### Incremental analysis

Comparison of the mean costs and mean QALYs associated with the strategy OMT with ICD vs the strategy OMT alone will be conducted using estimates from the long-term CEA model. An incremental cost-effectiveness ratio (ICER) will be calculated—if no strategy is found to be dominant^52^—to examine the additional costs required to achieve an additional QALY gain. In a budget-constrained healthcare system, having to pay additional costs to gain additional QALYs results in health lost somewhere else in the system, because funding one treatment necessarily shifts resources away from other uses, thereby forgoing their health benefits. In economic evaluation, the cost-effective treatment is therefore the one with the greatest health benefits, net of the health lost due to its costs. In practice, cost-effectiveness is assessed by comparing the ICER against a threshold whose value represents the rate at which the healthcare system improves health. Given the context of this evaluation is in the UK, where often policy decisions are made using the NICE threshold between £20 000/QALY and £30 000/QALY,^29^ we compare the ICER against this threshold as well as the cost-effectiveness threshold used by the Department of Health and Social Care in their impact assessments.^53 54^

### Subgroup analysis

If relevant, ICERs for different baseline risk profiles will be calculated to understand whether the ICD implantation with OMT can be more cost-effective for certain subgroups.^55 56^

### Sensitivity analyses

Sensitivity analyses will be conducted to explore the robustness of the study result with respect to the estimated uncertainties within the long-term CEA model. A one-way or multi-way sensitivity analysis will be performed on key deterministic parameters such as the discount rate and assumptions relating to the ICD device cost, device-related complications and the duration of treatment effects on overall mortality.

A probabilistic sensitivity analysis (PSA) will be conducted to reflect the sampling uncertainty in model parameter estimates using Monte Carlo simulation. The distributions of the model parameters (eg, transition probabilities or event rates, costs and EQ-5D) will be informed by the analysis of the trial data and external sources. The variance-covariance matrix for the sampling uncertainty of the parameters will be extracted from the regression models, and correlation between parameters in the regression models will be handled using the Cholesky decomposition method.^57^ A cost-effectiveness plane will be displayed to illustrate the distribution of incremental costs and incremental QALYs from the simulation. The results of the PSA will also be used to produce a cost-effectiveness acceptability curve to visualise the probability of cost-effectiveness across a range of cost-effectiveness thresholds.

Value of information analysis will be conducted to estimate the expected gain from reducing uncertainty through the collection of more information on key parameters and determine whether it is worth investing resources to obtain more information on these by funding further research in the future.^58 59^ Value of heterogeneity analysis will be performed to measure the gains from providing tailored recommendations across subgroups with current evidence and the value of future research to facilitate stratified treatment decisions.^60^

### Ethics and dissemination

The PROFID EHRA trial, under legal sponsorship of Charité—Universitätsmedizin Berlin, Germany, received its first ethics approval by the Medicine Research Ethics Committee of the La Paz University Hospital in Madrid, Spain (reference number LHS-2019-0209). Before including patients, for all participating study centres, the required local, central and/or national ethical approval has to be obtained. As of the date 13 November 2025, at least one participating study centre in the following countries has received ethical approvals from relevant ethics committees: Austria, Belgium, Czech Republic, Denmark, France, Germany, Great Britain, Hungary, Israel, the Netherlands, Poland and Spain. Results will be shared with the general public through various media channels and additionally with healthcare professionals and the scientific community through scientific meetings, conferences and publications.

## Discussion

The PROFID EHRA trial will reassess the effectiveness of ICD with OMT among post-MI patients with reduced LVEF, with a view to updating clinical guidance that is based on evidence from trials conducted more than two decades ago. The economic evaluation analysis proposed in this study will provide the cost and health implications of using OMT alone in place of ICD implantation on top of OMT, using existing evidence while the trial is ongoing and using the PROFID EHRA trial in combination with external sources on the availability of trial data.

The necessity of an economic evaluation in a non-inferiority trial might be questioned, especially if no mortality benefit is expected in the trial, and the health system can save money without compromising outcomes. However, conducting an economic evaluation in this scenario is still important for several reasons. First, the health effects and cost implications of ICD implantation can vary significantly among patients with different risk profiles.^55 56^ Understanding these variations can help develop tailored policy recommendations and ensure efficient resource allocation. Second, evidence from economic evaluation can provide comprehensive information about the magnitude of cost and benefit implications in the long term to support the implementation of policy changes. This includes not only direct short-term savings but also potential shifts in resource allocation in the longer term as patients’ earlier treatment pathway modifies their need for subsequent care. Lastly, the results of economic evaluation can reflect trade-offs that may be observed in the final results. For instance, even if the trial reveals slightly worse clinical outcomes but with significantly reduced costs, it may still be considered cost-effective as the cost savings can be invested by payers to support other forms of care in the system. The decision-makers will then need to trade off between the health losses in the clinical population in focus and the health gains achievable by freeing up resources to spend on other healthcare priorities. Thus, an economic evaluation is essential for informed, nuanced decision-making in non-inferiority trials such as PROFID EHRA.

This proposed economic evaluation focuses on ischaemic patients, in line with the inclusion criteria of the PROFID EHRA trial.^21^ Non-ischaemic cardiomyopathy is not considered as their risk stratification and potential benefit from ICD therapy has been studied in the Danish Study to Assess the Efficacy of ICDs in Patients with Non-ischemic Systolic Heart Failure on Mortality (DANISH) which found no significant reduction in long-term all-cause mortality of ICD implantation compared with OMT.^61^ As such, the findings from this study cannot be transferred to the non-ischaemic population, where the clinical and economic value of ICDs remains an important but distinct question.

One strength of this economic evaluation protocol is that it proposes an evidence-based iterative approach for evaluating the cost-effectiveness of ICD implantation for post-MI patients with reduced LVEF. The long-term model-based pre-trial CEA will provide evidence to support potential disinvestment decisions about the use of ICDs in the patient population defined by the PROFID EHRA trial and quantify the extent to which further research in this decision space is required. The within-trial CCA and long-term model-based post-trial CEA will benefit from the direct measurement of both health outcomes and resource use within the same study context, providing robust evidence for decision-making in an RCT setting instead of using data solely from external sources.

The limitations of this study are mainly attributed to uncertainties in model structure and model parameters. The proposed CEA model structure will consider two health states (ie, Alive and All-cause death), which differs from some previous published studies^62^,^67^ that considered specific causes of death in the CEA. The reason for not specifying the death causes in the proposed model is that the all-cause mortality is the primary endpoint of the PROFID EHRA trial,^21^ and it is preferred that the health states in the long-term CEA model align with the trial design. To reflect that the purpose of ICD implantation is to reduce risk of SCD, an additional model structure that includes both SCD and Death due to other causes will be considered in the sensitivity analysis upon the availability of trial data. The transient events are not assumed to structurally affect all-cause mortality, as the all-cause mortality for OMT alone and ICD+OMT will reflect the fact that some patients receiving ICD will experience complications and replacements which may result in death, and modelling their impacts on mortality may add unnecessary structural complexity and introduce the risk of double counting.^68^

In addition, for the pre-trial CEA, several model parameters will need to be derived from the published literature and/or experts’ opinion; and even for the post-trial CEA, given the limited trial observation period, some potentially important parameters will remain uncertain such as the duration of the treatment effect on mortality, long-term rates of device replacement, etc. The risk of complications related to ICD devices may vary by device type,^17^ and depending on data quality in the trial, the impact of choice of device will be explored using sensitivity analysis.

This economic evaluation applies an NHS and PSS perspective as recommended by NICE guidance in the UK,^29^ with a view to extending evaluation to the contexts of other participating countries in due course. We consider that the proposed model structure will generalise to other healthcare systems. However, the application of this economic evaluation protocol to other specific countries’ contexts may not be straightforward, depending on multiple factors including quantity and quality of the data collection in the country of interest, whether this country participated in the PROFID EHRA trial, as well as the normative framework for using economic evaluation studies to guide health policy decisions in each jurisdiction.

From a societal perspective,^69^ the indirect costs such as out-of-pocket and productivity losses, as well as the environmental impacts of device manufacturing, replacement and end-of-life disposal, may not affect both treatment strategies equally. While a societal perspective is beyond the scope of this evaluation study, an explicit consideration of these additional costs (and benefits) will be necessary to inform funding decisions in those jurisdictions that mandate a broader perspective than the one adopted in the UK.

In conclusion, this protocol outlines the economic evaluation methods to assess the value for money of using OMT alone in place of currently recommended treatment—ICD with OMT—among post-MI patients with reduced LVEF. Its findings will provide important and up-to-date insights for shaping policy decisions regarding the recommendation of ICD implantation in this patient population.
