# Supplementary material for: Economic evaluation protocol for the PRevention Of sudden cardiac death aFter myocardial Infarction by Defibrillator implantation: the PROFID EHRA trial
Source: BMJ Open. 2026 Jan 8;16(1):e097495. doi: 10.1136/bmjopen-2024-097495 (PMC13059845; doi:10.1136/bmjopen-2024-097495)
Supplement: Supplementary file 1 [file bmjopen-2024-097495.reviewer_comments.pdf]

PEER REVIEW HISTORY

BMJ Open publishes all reviews undertaken for accepted manuscripts. Reviewers are asked to complete a checklist review form and are provided with free text boxes to elaborate on their assessment. These free text comments are reproduced below.

ARTICLE DETAILS

Title (Provisional)

Economic evaluation protocol for the PRevention Of sudden cardiac death aFter myocardial Infarction by Defibrillator implantation: the PROFID EHRA trial

Authors

Qian, Yirui; Roque, Carlos Rojas; Woods, B; Iglesias, C; Gc, Vijay S.; Gur Arie, Maria; Fischer, Daniela; Dagres, Nikolaos; Hindricks, Gerhard; Manca, Andrea

---

VERSION 1 - REVIEW

**Reviewer**	**1**
**Name**	**Quayyum , Zahidul**
**Affiliation**	**BRAC University, BRAC James P Grant School of Public Health**
**Date**	**15-Feb-2025**
**COI**	**None**

---

1. As regards statement of the problem or in the Background/ introduction section the authors need to mention about the extent of the problem, i.e. what proportion of the patient or what is incidence sudden cardiac death (SCD) in the population with post myocardial infarction. Not clear - though line 5 and 4. on page page noted that - not sure whether this is among the population with post myocardial with reduced left ventricular ejection fraction (LVEF).

4. Methods section : the model and data analysis plan is well explained, however,

the authors need to mention - how far the recruitment of the patient has been completed at the time of submission of the manuscript.

It has been mentioned that the total number of patient recruited will be 3595 in 180 countries in Europe and in Israel. Can the author mention how this number has been reached. I understand the total number is based on the sample size estimation to study the effectiveness of the clinical intervention, however, this can be mentioned.

One more issue regarding the patient recruitment is regarding :

Will all the patients recruited are with reduced LVEF ≤ 35%, i.e. the reduced LVEF is strictly with this value, if so this has to always refer to - not just mention with reduced LVEF.

Besides, this is a multicounty study, the name of the countries in Europe - can mentioned here. This is an useful information that has been omitted. Or you can mentioned that further detail are available in (ref?).

In the methods section, the resource use and costs section section (2.4.2 page 7 and 8), the authors suggests they will use UK data :

They mention that "Unit cost data will be obtained from national data sources such as the British National Formulary, the PSS Research Unit, the National cost collection for NHS. What about for other European countries and Israel - what would be the source of data for the unit costs of resources needed .

Given the above comments the limitation section may need to be revised.

5. As regards Research ethics - I did not find specific mention about ethical approval, and probably this is needed in different project participating countries.

**Reviewer**	**2**
**Name**	**van Barreveld, Marit**
**Affiliation**	**Amsterdam UMC Locatie AMC, Epidemiology and Data Science**
**Date**	**14-Jul-2025**
**COI**	**None**

This is a clearly written protocol that outlines the economic evaluation planned alongside the PROFID EHRA trial. The relevance of this study is extremely high, from both a clinical and economic perspective. As clinical guidelines for ICD use are still largely based on evidence from decades-old trials, the PROFID EHRA trial – and its embedded economic evaluation – address a long-standing gap in contemporary evidence. The field is eagerly awaiting the results of this trial, and the authors are to be commended for their contribution to such an important and timely topic.

The protocol describes a robust and comprehensive approach to both within-trial cost-consequence analysis and long-term cost-effectiveness modelling. Nevertheless, a number of clarifications and refinements are suggested below to enhance transparency, validity, and applicability of the analysis.

Specific Comments

1. The study includes only post-myocardial infarction (ischaemic) patients with reduced LVEF. This exclusion is not addressed or justified in the introduction or discussion. Given that the clinical benefit of ICD implantation in non-ischaemic cardiomyopathy patients has been even more debated in recent years, one could argue that this group would have been particularly relevant for inclusion in a contemporary economic evaluation. While the decision may be deliberate, the protocol would benefit from a brief justification and a reflection on how this affects the applicability of the findings

2. The manuscript does not define what constitutes a device-related complication. Are both major and minor complications included? Furthermore, it is unclear whether inappropriate ICD shocks are included as complications, and whether their associated costs and impact on quality of life are captured in the model.

3. Clarification is needed on whether all included ICDs are single- or dual-chamber, or include CRT-D devices. For patients who are randomised to OMT but meet criteria for CRT-D, will CRT-P devices be provided? If so, are pacemaker-related costs and complications accounted for in the analysis? Additionally, does the risk of complications (following replacement) vary by ICD type? if so, it would be important to reflect this heterogeneity in the long-term modelling, as it could influence both cost and quality of life outcomes.

4. Although the authors assume that transient events do not structurally affect all-cause mortality, evidence from the DO-IT study (e.g. 5 out of 1,443 patients with complication-related death) and DAI-PP Registry suggests that this may not be entirely appropriate. The authors should consider incorporating this risk into sensitivity analyses or justify its exclusion more explicitly.

5. The evaluation uses an NHS and PSS perspective but does not mention costs incurred outside the hospital (e.g., GP visits, ambulance services, or rehabilitation). These are particularly relevant for patients in the OMT arm who may experience out-of-hospital cardiac arrest. This limitation should be explicitly acknowledged, along with a justification.

6. The evaluation does not incorporate a societal perspective. Out-of-pocket costs and productivity losses, especially relevant when experiencing transient events, are excluded. This omission could bias the analysis in favour of ICDs. The authors should reflect on this limitation and discuss the potential impact on cost-effectiveness conclusions.

7. The lifetime horizon is mentioned but not defined. Will modelling continue until all patients are projected to have died, or is there an upper age limit? A clear specification is recommended.

8. In long-term non-inferiority trials, patients in the OMT arm may receive ICDs later due to clinical deterioration. How will such crossovers be handled in the economic analysis? This should be discussed in the methods section.

9. The economic model applies UK EQ-5D tariffs and adopts an NHS/PSS perspective, both of which are consistent with NICE guidance. However, the trial is being conducted across

multiple European countries. The authors should clarify whether the distribution of patient recruitment across participating countries has been assessed and whether this might influence utility estimates and cost assumptions.

Using UK-specific utility tariffs and unit costs may limit the generalisability of the findings to non-UK settings, particularly if a substantial proportion of trial participants is recruited from outside the UK. In addition, healthcare costs may vary significantly across healthcare systems. Even though this analysis is appropriately focused on the UK context, the authors may wish to acknowledge this limitation more explicitly and indicate whether future country-specific or cross-jurisdictional cost-effectiveness analyses are planned using the trial data. Such a clarification would enhance the transparency of the analysis and increase its relevance to decision-makers beyond the UK.

10. The authors rightly argue that an economic evaluation remains valuable in a non-inferiority trial, particularly to assess scenarios where cost savings may be achieved at the expense of marginal health losses. While this point is touched upon in the discussion, further elaboration would strengthen the manuscript. Specifically, how will such trade-offs (slightly worse clinical outcomes but substantially lower costs) be communicated to policymakers? What level of clinical inferiority is considered acceptable if it leads to a more efficient use of healthcare resources? Moreover, while the analysis adopts the commonly used UK cost-effectiveness threshold of £20,000–£30,000 per QALY, this may not fully capture the ethical dimension of potentially de-implementing an established therapy. In such cases, some argue that a willingness-to-accept (WTA) threshold (reflecting society's reluctance to accept health losses even in the face of cost savings) may be more appropriate than the standard willingness-to-pay (WTP) threshold. The authors are encouraged to reflect on whether this distinction is relevant to their analysis, and how it might influence the interpretation of their findings in the context of policy recommendations.

11. As environmental impact becomes an increasingly important consideration in healthcare policy, it may be worth reflecting on the potential sustainability implications of different treatment strategies. Implantable devices such as ICDs involve resource-intensive manufacturing, periodic replacement, and end-of-life disposal. Although this falls outside the traditional scope of health economic evaluations, have the authors considered whether environmental sustainability (such as carbon footprint or material use) could be included as an exploratory argument or in future extensions of this work?

VERSION 1 - AUTHOR RESPONSE

Reviewer: 1
Prof. Zahidul Quayyum , BRAC University
Comments to the Author:

**Reviewer Comment**	**Response / Action**	**Location in Manuscript (Clean version)**
Comment 1: 1. As regards statement of the problem or in the Background/ introduction section the authors need to mention about the extent of the problem, i.e. what proportion of the patient or what is incidence sudden cardiac death (SCD) in the population with post myocardial infarction. Not clear - though line 5 and 4. on page page noted that - not sure whether this is among the population with post myocardial with reduced left ventricular ejection fraction (LVEF).	Thanks for your suggestion. We have added this information in the backgrounds	1 Background<br>Page 4 line 3-6<br>*'It is reported that the overall incidence of SCD for patients who have survived a myocardial infarction (MI) ranges from 2% to 4% per year, with significantly increased risk within the first few months post MI* [1]. *Coronary artery disease (CAD) is believed to be responsible for about 3/4 of SCD cases* [2].*'*
Comment 2: 4. Methods section: the model and data analysis plan is well explained, however, the authors need to mention - how far the recruitment of the patient has been completed at the time of submission of the manuscript. It has been mentioned that the total number of patient recruited will be 3595 in 180 countries in Europe and in Israel. Can the author mention how this number has been reached. I understand the total number is based on the sample size estimation to study the effectiveness of the clinical intervention, however, this can be mentioned.	Thanks for this comment. The determination of sample size and up-to-date recruitment is clearly justified in the recently published protocol [3], which is mentioned and cited under 2.1 PROFID EHRA trial design. We agree that clarification is needed for determined sample size, and we have improved this section to reflect this. We have also included the trial recruitment as per the trial published protocol [3].	2.1 PROFID EHRA trial design<br>Page 5 line 9-11<br>*'The total sample size of 3,595 patients was determined by the responsible PROFID EHRA statistician based on formal sample size calculations* [3]. *As of 20 October 2025, 256 of the planned 3,595 patients had been enrolled in 83 of 180 planned participating sites* [3]. *'*
Comment 3: One more issue regarding the patient recruitment is regarding : Will all the patients recruited are with reduced LVEF ≤ 35%, i.e. the reduced LVEF is strictly with this value, if so this has to always refer to - not just mention with reduced LVEF.	All patients recruited into the PROFID EHRA trial are required to have LVEF of ≤ 35% as part of the inclusion criteria. To improve clarity and consistency, we have revised the manuscript to state explicitly that reduced LVEF is defined as LVEF ≤ 35% and that this definition will be used consistently throughout the text.	2.1 PROFID EHRA trial design<br>Page 5 line 5-7<br>*'The trial is event-driven to test whether OMT alone is not inferior to OMT with ICD implantation in post-MI patients with LVEF ≤ 35% (referred to here as 'reduced LVEF') with respect to all-cause mortality within 2.5 years of observation post index-date.'*
Comment 4: Besides, this is a multicounty study, the name of the countries in Europe - can mentioned here. This is an useful information that has been omitted. Or you can	Thank you for this suggestion. We have not listed all countries in the main text to maintain conciseness, but a full list of participating countries is available the clinical trial registry website [4], which is	2.1 PROFID EHRA trial design<br>Page 5 line 14-15<br>*'Study details of the PROFID EHRA trial design are*

Reviewer Comment	Response / Action	Location in Manuscript (Clean version)
mentioned that further detail are available in (ref?).	cited under '2.1 PROFID EHRA trial design'.	*published in the study protocol of the trial* [3] *and on the clinical trial registry website* [4].*'*
Comment 5: In the methods section, the resource use and costs section section (2.4.2 page 7 and 8), the authors suggests they will use UK data :<br>They mention that "Unit cost data will be obtained from national data sources such as the British National Formulary, the PSS Research Unit, the National cost collection for NHS. What about for other European countries and Israel - what would be the source of data for the unit costs of resources needed .<br>Given the above comments the limitation section may need to be revised.	The economic evaluation will be conducted from the perspective of the UK NHS and PSS. The perspective of the analysis determines how resource uses are costed, which is why we apply the UK unit cost data. Conducting the evaluation from other participating countries' perspective will be considered, with its feasibility depending on the quantity and quality of the data collected by those countries and the relevance to their specific economic evaluation frameworks. We have explained this in the discussion section.	<u>4 Discussion</u><br>Page 11 line 36-43<br>*'This economic evaluation applies an NHS and PSS perspective as recommended by NICE guidance in the United Kingdom* [5], *with a view of extending evaluation to the contexts of other participating countries in due course. We consider that the proposed model structure will generalise to other health care systems. However, the application of this economic evaluation protocol to other specific countries' context may not be straightforward, depending on multiple factors including quantity and quality of the data collection in the country of interest, whether this country participated in the PROFID EHRA trial, as well as the normative framework for using economic evaluation studies to guide health policy decisions in each jurisdiction.'*
Comment 6: 5. As regards Research ethics - I did not find specific mention about ethical approval, and probably this is needed in different project participating countries.	Thanks for this comment. We have added a section in the main text, named '3.8 Ethics and dissemination'.	<u>3.8 Ethics and dissemination</u><br>Page 10

Reviewer: 2
Dr. Marit van Barreveld, Amsterdam UMC Locatie AMC

Reviewer Comment	Response / Action	Location in Manuscript (Clean version)
Comments to the Author:		

**Reviewer Comment**	**Response / Action**	**Location in Manuscript (Clean version)**
This is a clearly written protocol that outlines the economic evaluation planned alongside the PROFID EHRA trial. The relevance of this study is extremely high, from both a clinical and economic perspective. As clinical guidelines for ICD use are still largely based on evidence from decades-old trials, the PROFID EHRA trial – and its embedded economic evaluation – address a long-standing gap in contemporary evidence. The field is eagerly awaiting the results of this trial, and the authors are to be commended for their contribution to such an important and timely topic.	We sincerely thank the reviewer for these positive and encouraging comments regarding the relevance and importance of our study.	No change needed
The protocol describes a robust and comprehensive approach to both within-trial cost-consequence analysis and long-term cost-effectiveness modelling. Nevertheless, a number of clarifications and refinements are suggested below to enhance transparency, validity, and applicability of the analysis.	We thank the reviewer for their positive assessment of our approach and for the constructive suggestions provided. We have addressed each of the points raised below to enhance the clarity, transparency, and applicability of the protocol.	No change needed
Specific Comments		
Comment 1. The study includes only post-myocardial infarction (ischaemic) patients with reduced LVEF. This exclusion is not addressed or justified in the introduction or discussion. Given that the clinical benefit of ICD implantation in non-ischaemic cardiomyopathy patients has been even more debated in recent years, one could argue that this group would have been particularly relevant for inclusion in a contemporary economic evaluation. While the decision may be deliberate, the protocol would benefit from a brief justification and a reflection on how this affects the applicability of the findings	The PROFID EHRA trial specifically focuses on patients with ischaemic cardiomyopathy post-myocardial infarction, as this population is considered at higher risk of sudden cardiac death and represents the target group for the intervention under evaluation. Patients with non-ischaemic cardiomyopathy are outside the scope of this trial, and therefore not considered in this economic evaluation. We have revised the Discussion to justify this focus and to acknowledge that the exclusion of non-ischaemic cardiomyopathy patients may limit the generalisability of the findings to that population.	<u>Discussion</u><br>Page 11 line 4 – 9<br>*'This proposed economic evaluation focuses on ischaemic patients, in line with the inclusion criteria of the PROFID EHRA trial* [3]. *Non-ischaemic cardiomyopathy is not considered as their risk stratification and potential benefit from ICD therapy has been studied in the DANISH trial which found no significant reduction in long-term all-cause mortality of ICD implantation compared to OMT* [6]. *As such, the findings from this study cannot be transferred to the non-ischaemic population, where the clinical and economic value of ICDs*

Reviewer Comment	Response / Action	Location in Manuscript (Clean version)
		remains an important but distinct question.'
Comment 2. The manuscript does not define what constitutes a device-related complication. Are both major and minor complications included? Furthermore, it is unclear whether inappropriate ICD shocks are included as complications, and whether their associated costs and impact on quality of life are captured in the model.	Device-related complications are defined and listed in the Methods section, as validated by clinical experts. Both major and minor complications are included. A sensitivity analysis will be considered for looking at potential implications of inappropriate shocks.	3.3.2 Modelling transient events<br>Page 8 line 33-35<br>'*The following device-related complications will be considered for the analysis: lead failure, tamponade, pneumothorax, dislocation of lead, significant pocket hematoma and infection, as validated by clinical experts from the PROFID EHRA trial.*'<br><br>3.7 Sensitivity analyses<br>Page 9 line 35-38<br>*'A one-way or multi-way sensitivity analysis will be performed on key deterministic parameters such as the discount rate and assumptions relating to the ICD device cost, device-related complications, and the duration of treatment effects on overall mortality.'*
Comment 3. Clarification is needed on whether all included ICDs are single- or dual-chamber, or include CRT-D devices. For patients who are randomised to OMT but meet criteria for CRT-D, will CRT-P devices be provided? If so, are pacemaker-related costs and complications accounted for in the analysis? Additionally, does the risk of complications (following replacement) vary by ICD type? if so, it would be important to reflect this heterogeneity in the long-term modelling, as it could influence both cost and quality of life outcomes.	Patients who are eligible for CRT are excluded from the trial. The type of ICD device used (including whether subcutaneous or transvenous) is at the discretion of the investigator, and all aspects of device implantation. This detail is reported in the trial published protocol and cited under '2.1 PROFID EHRA trial design'.<br>We acknowledge that the risk of complications may vary by device type, and the extent to which we can explore its role in affecting the costs and QALYs in the intervention group (and the resulting impact on the ICER) will depend on the frequency of use of the different types of device, which will only become clear when the trial data become available. We have added further details to the manuscript to clarify these points.	4 Discussion<br>Page 11 line 33-35<br>*'The risk of complications related to ICD device may vary by device type [17], and depending on data quality in the trial, the impact of choice of device will be explored using sensitivity analysis.'*

Reviewer Comment	Response / Action	Location in Manuscript (Clean version)
Comment 4. Although the authors assume that transient events do not structurally affect all-cause mortality, evidence from the DO-IT study (e.g. 5 out of 1,443 patients with complication-related death) and DAI-PP Registry suggests that this may not be entirely appropriate. The authors should consider incorporating this risk into sensitivity analyses or justify its exclusion more explicitly.	The survival analysis of all-cause mortality reflects the net effect of ICD therapy and its transient events. We don't therefore assume that transient events do not structurally affect all-cause mortality, but instead that the relationship between transient events and all-cause mortality is already reflected in all-cause mortality outcomes. This is now explained in the discussion section.	4 Discussion<br>Page 11 Line 25-29<br>*'The transient events are not assumed to structurally affect all-cause mortality, as the all-cause mortality for ICD and OMT will reflect the fact that some patients receiving ICD will experience complications and replacements which may result in death, and modelling their impacts on mortality may add unnecessary structural complexity and introduce the risk of double counting* [7]*'*
Comment 5. The evaluation uses an NHS and PSS perspective but does not mention costs incurred outside the hospital (e.g., GP visits, ambulance services, or rehabilitation). These are particularly relevant for patients in the OMT arm who may experience out-of-hospital cardiac arrest. This limitation should be explicitly acknowledged, along with a justification.	Resource use outside the hospital setting (e.g., GP visits, ambulance services, or rehabilitation) is not collected within the trial. While these costs are relevant from an NHS and PSS perspective, we do not expect significant differences for the occurrence of non-fatal events outside hospital setting in both treatment strategies. The main cost will be accumulated within the hospital rather than the community. We have improved the discussion session to reflect this.	Discussion<br>Page 12 Line 1-4<br>*'Although resource use outside the hospital setting (e.g. visits to general practitioner, ambulance services, or rehabilitation) are not captured in the analysis due to data availability, the occurrence of non-fatal events is not expected to differ significantly in both arms of the trial* [8]*, and the main cost is expected to accumulate the most in the hospital rather than the community.'*
Comment 6. The evaluation does not incorporate a societal perspective. Out-of-pocket costs and productivity losses, especially relevant when experiencing transient events, are excluded. This omission could bias the analysis in favour of ICDs. The authors should reflect on this limitation and discuss the potential impact on cost-effectiveness conclusions.	Our evaluation adopts the NHS and PSS perspective, consistent with NICE guidance, and therefore does not include out-of-pocket costs or productivity losses.<br><br>In any case, we are unclear whether their inclusion would bias the analysis in favour of ICDs. It is ultimately an empirical question. We have revised the discussion to reflect this point.	4 Discussion<br>Page 12 line 4-7<br>*'Furthermore, a societal perspective to quantify the impact an intervention may have on indirect costs such as out-of-pocket and productivity losses, as well as the environmental impacts of device manufacturing, replacement, and end-of-life disposal [69] are beyond the scope of this evaluation study.'*

**Reviewer Comment**	**Response / Action**	**Location in Manuscript (Clean version)**
Comment 7. The lifetime horizon is mentioned but not defined. Will modelling continue until all patients are projected to have died, or is there an upper age limit? A clear specification is recommended.	We agree there needs to be some clarification regarding the lifetime horizon. This is defined as continuing until all patients in the simulated cohort are projected to have died, with no imposed upper age limit. We have clarified this definition in the revised manuscript.	<u>2.3 Economic evaluation methods</u><br>Page 6 line 14-15<br>*'The CEA models take a lifetime horizon that projects patient outcomes over their entire life course till death.'*
Comment 8. In long-term non-inferiority trials, patients in the OMT arm may receive ICDs later due to clinical deterioration. How will such crossovers be handled in the economic analysis? This should be discussed in the methods section.	As specified in the published trial protocol [3], if patients randomised to the non-ICD arm subsequently experience ventricular arrhythmias requiring secondary prevention ICD implantation, this is not considered a crossover but rather a potential consequence of the initial no-ICD strategy. Our economic evaluation will follow this definition: secondary prevention ICD implantations in the OMT arm will be treated as downstream clinical events, with their associated costs and outcomes captured accordingly. We have clarified this in the Methods section.	<u>2.1 PROFID EHRA trial design</u><br>Page 5 line 11-15<br>*'Secondary prevention ICD implantations in patients initially randomised to the OMT arm are considered as a potential 'real world' consequence of the initial strategy rather than crossovers* [3]*, which is consistent with the trial intention-to-treat analysis. Study details of the PROFID EHRA trial design are published in the study protocol of the trial* [3] *and on the clinical trial registry website* [4]*.'*
Comment 9. The economic model applies UK EQ-5D tariffs and adopts an NHS/PSS perspective, both of which are consistent with NICE guidance. However, the trial is being conducted across multiple European countries. The authors should clarify whether the distribution of patient recruitment across participating countries has been assessed and whether this might influence utility estimates and cost assumptions.<br>Using UK-specific utility tariffs and unit costs may limit the generalisability of the findings to non-UK settings, particularly if a substantial proportion of trial participants is recruited from outside the UK. In addition, healthcare costs may vary significantly across healthcare systems. Even though this analysis is appropriately focused on the UK context, the authors may wish to acknowledge this limitation more explicitly and indicate	At present, we are unable to provide information on the distribution of patient recruitment across participating countries, as the trial is ongoing and recruitment data are not publicly disclosed to protect trial integrity.<br>The economic evaluation is designed to reflect the context of the UK healthcare system, applying UK-specific EQ-5D utility tariffs and NHS/PSS unit costs, in line with NICE guidance. We acknowledge that this choice may limit somewhat the generalisability of cost and utility estimates to non-UK settings, given variations in healthcare costs and systems across countries. As each economic evaluation must reflect the context and data of the individual jurisdiction, country-specific or cross-jurisdictional cost-effectiveness	<u>Discussion</u><br>Page 11 line 36-43<br>*'This economic evaluation applies an NHS and PSS perspective as recommended by NICE guidance in the United Kingdom* [5]*, with a view of extending evaluation to the contexts of other participating countries in due course. We consider that the proposed model structure will generalise to other health care systems. However, the application of this economic evaluation protocol to other specific countries' context may not be straightforward, depending on multiple factors including quantity and quality of the data*

Reviewer Comment	Response / Action	Location in Manuscript (Clean version)
whether future country-specific or cross-jurisdictional cost-effectiveness analyses are planned using the trial data. Such a clarification would enhance the transparency of the analysis and increase its relevance to decision-makers beyond the UK.	analyses are not planned as part of this evaluation. We have improved this clarification in the Discussion section.	*collection in the country of interest, whether this country participated in the PROFID EHRA trial, as well as the normative framework for using economic evaluation studies to guide health policy decisions in each jurisdiction. '*
Comment 10. The authors rightly argue that an economic evaluation remains valuable in a non-inferiority trial, particularly to assess scenarios where cost savings may be achieved at the expense of marginal health losses. While this point is touched upon in the discussion, further elaboration would strengthen the manuscript. Specifically, how will such trade-offs (slightly worse clinical outcomes but substantially lower costs) be communicated to policymakers? What level of clinical inferiority is considered acceptable if it leads to a more efficient use of healthcare resources? Moreover, while the analysis adopts the commonly used UK cost-effectiveness threshold of £20,000–£30,000 per QALY, this may not fully capture the ethical dimension of potentially de-implementing an established therapy. In such cases, some argue that a willingness-to-accept (WTA) threshold (reflecting society's reluctance to accept health losses even in the face of cost savings) may be more appropriate than the standard willingness-to-pay (WTP) threshold. The authors are encouraged to reflect on whether this distinction is relevant to their analysis, and how it might influence the interpretation of their findings in the context of policy recommendations.	We are glad that the reviewer agrees that economic evaluation in a non-inferiority trial provides important insights into scenarios where cost savings may be achieved at the expense of marginal health losses. As stated in our protocol, trade-offs between small reductions in clinical outcomes and potential cost savings will be communicated using incremental cost-effectiveness ratios (ICER), cost-effectiveness acceptability plane (CEAP), and cost-effectiveness acceptability curves, highlighting the probability that each strategy is cost-effective at different willingness-to-pay thresholds, following NICE guidance. We have expanded the discussion to highlight the interpretation of results using net health benefits that allows the trade-off between potential health losses in the trial population and health gains that could be achieved by reallocating resources to other healthcare priorities.<br><br>The clinical inferiority of 0.75 (ICD+OMT vs OMT alone) is stated in the recent published trial protocol [3]. We agree that WTA considerations may influence policy interpretation, particularly when evaluating strategies that reduce established therapy use (i.e. south-west quadrant in the CEAP). However, there is currently no published guidance on WTA and we wouldn't be able to gather this information. We are going to present all available evidence following the	<u>Discussion</u><br>Page 10 line 41- Page 11 line 1<br>*'For instance, even if the trial reveals slightly worse clinical outcomes but with significantly reduced costs, it may still be considered cost-effective as the cost savings can be invested by payers to support other forms of care in the system. The decision makers will then need to trade-off between the health losses in the clinical population in focus and the health gains achievable by freeing up resources to spend on other health care priorities.'*

**Reviewer Comment**	**Response / Action**	**Location in Manuscript (Clean version)**
	NICE guidance to policy makers for them to decide.	
Comment 11. As environmental impact becomes an increasingly important consideration in healthcare policy, it may be worth reflecting on the potential sustainability implications of different treatment strategies. Implantable devices such as ICDs involve resource-intensive manufacturing, periodic replacement, and end-of-life disposal. Although this falls outside the traditional scope of health economic evaluations, have the authors considered whether environmental sustainability (such as carbon footprint or material use) could be included as an exploratory argument or in future extensions of this work?	We appreciate this thoughtful comment. While we recognise the growing importance of environmental sustainability in healthcare decision-making, this analysis was designed to reflect the NHS and PSS perspective, in line with NICE reference case requirements. Broader considerations, e.g. environmental impact of device manufacturing, replacement, and disposal, would fall under a wider societal perspective, which is outside the scope of the current evaluation. However, we acknowledge that incorporating sustainability dimensions into future economic evaluations could provide valuable insights for policy decisions, particularly as relevant data and methodological frameworks continue to develop.	<u>Discussion</u><br>Page 12 Line 4-7<br>*'Furthermore, a societal perspective to quantify the impact an intervention may have on indirect costs such as out-of-pocket and productivity losses, as well as the environmental impacts of device manufacturing, replacement, and end-of-life disposal* [9] *are beyond the scope of this evaluation study.'*

Reference list:

1 Zaman S, Kovoor P. Sudden Cardiac Death Early After Myocardial Infarction. *Circulation*. 2014;129:2426–35. doi: 10.1161/CIRCULATIONAHA.113.007497

2 Zeppenfeld K, Tfelt-Hansen J, de Riva M, *et al.* 2022 ESC Guidelines for the management of patients with ventricular arrhythmias and the prevention of sudden cardiac death: Developed by the task force for the management of patients with ventricular arrhythmias and the prevention of sudden cardiac death of the European Society of Cardiology (ESC) Endorsed by the Association for European Paediatric and Congenital Cardiology (AEPC). *European Heart Journal*. 2022;43:3997–4126. doi: 10.1093/eurheartj/ehac262

3 Dagres N, Gale CP, Nadarajah R, *et al.* PRevention Of sudden cardiac death aFter myocardial Infarction by Defibrillator im-plantation: Design and Rationale of the PROFID EHRA randomised clinical trial. *American Heart Journal*. Published Online First: 6 August 2025. doi: 10.1016/j.ahj.2025.07.071

4 Køber L, Thune JJ, Nielsen JC, *et al.* Defibrillator Implantation in Patients with Nonischemic Systolic Heart Failure. *New England Journal of Medicine*. 2016;375:1221–30. doi: 10.1056/NEJMoa1608029

5 Prevention Of Sudden Cardiac Death After Myocardial Infarction by Defibrillator Implantation (PROFID EHRA). ClinicalTrials.gov. 2024. https://clinicaltrials.gov/study/NCT05665608

6 van Barreveld M, Verstraelen TE, van Dessel PFHM, *et al.* Dutch Outcome in Implantable Cardioverter-Defibrillator Therapy: Implantable Cardioverter-Defibrillator–Related Complications in a Contemporary Primary Prevention Cohort. *Journal of the American Heart Association*. 2021;10:e018063. doi: 10.1161/JAHA.120.018063

7 National Institute for Health and Care Excellence. *NICE health technology evaluations: the manual*. London: National Institute for Health and Care Excellence 2022.

VERSION 2 - REVIEW

Reviewer 2

Name van Barreveld, Marit

Affiliation Amsterdam UMC Locatie AMC, Epidemiology and Data Science

Date 27-Nov-2025

COI

Thank you very much for the thorough revision of the manuscript and for addressing the reviewer comments in detail. I appreciate the improvements made throughout the paper and I am overall satisfied with the changes.

I have one remaining comment regarding the response to Comment 6, specifically the discussion around productivity costs. I understand that, given the chosen NHS and PSS perspective, productivity losses will not be included in the economic evaluation. My intention is not to request their inclusion, but simply to note that these effects are unlikely to be neutral.

In my view, ICD implantation is likely to lead to higher productivity losses than OMT alone. ICD recipients may require time off work for the implantation procedure, and for potential device-related complications, inappropriate shocks, or additional outpatient visits or hospitalisations. Although the magnitude of this effect is uncertain and may ultimately be small given the characteristics of the study population, the direction of the impact is reasonably predictable. A similar argument applies to the environmental impacts associated with device manufacturing, replacement, and end-of-life disposal, which are also unlikely to be evenly distributed across study arms.

I agree that a protocol paper is not the place to conduct or fully explore these additional perspectives. However, when discussing limitations, it might be helpful to clarify that the exclusion of these elements (productivity losses and environmental impacts) may influence the interpretation of cost-effectiveness results. If the authors consider this reasoning appropriate, they might also consider slightly refining the relevant sentence in the discussion to reflect that these factors may not affect both arms equally and may warrant more explicit consideration when reporting the full trial results.

You may wish to consider rephrasing it along the lines of:

“While a societal perspective (including consideration of productivity losses, out-of-pocket expenses, and environmental impacts) is beyond the scope of this evaluation study, these factors may not affect both trial arms equally. Their exclusion should therefore be recognised

as a limitation and may warrant more explicit consideration when interpreting and reporting the full trial results."

Thank you again for the careful revisions and for the opportunity to review this interesting and important protocol. I look forward to seeing the results of the study.

VERSION 2 - AUTHOR RESPONSE

Reviewer: 2
Dr. Marit van Barreveld, Amsterdam UMC Locatie AMC

Reviewer Comment	Response	Location in Manuscript (Clean version)
Thank you very much for the thorough revision of the manuscript and for addressing the reviewer comments in detail. I appreciate the improvements made throughout the paper and I am overall satisfied with the changes.	We sincerely thank the reviewer for this positive and encouraging comment regarding our revised manuscript.	No change needed
I have one remaining comment regarding the response to Comment 6, specifically the discussion around productivity costs. I understand that, given the chosen NHS and PSS perspective, productivity losses will not be included in the economic evaluation. My intention is not to request their inclusion, but simply to note that these effects are unlikely to be neutral.	Thank you for this clarification. We agree that productivity losses for each treatment arm may not be the same.	Please see changes in the last response
In my view, ICD implantation is likely to lead to higher productivity losses than OMT alone. ICD recipients may require time off work for the implantation procedure, and for potential device-related complications, inappropriate shocks, or additional outpatient visits or hospitalisations. Although the magnitude of this effect is uncertain and may ultimately be small given the characteristics of the study population, the direction of the impact is reasonably predictable. A similar argument applies to the environmental impacts associated with device manufacturing, replacement, and end-of-life disposal, which are also unlikely to be evenly distributed across study arms.	We appreciate the insights shared by the reviewer, and we agree that the patients with ICD may require time-off for the implantation procedure and may face potential device-related complications or inappropriate shocks that could lead to productivity loss. However, patients on OMT arm may also bear productivity loss due to ventricular arrhythmia that may not be promptly detected and treated as patients on ICD arm. We agree that the ICD arm may incur additional environmental impacts associated with device manufacturing, replacement, and end-of-life disposal.<br><br>We think it remains uncertain how the cost-effectiveness results could change when considering additional	Please see changes in the last response

**Reviewer Comment**	**Response**	**Location in Manuscript (Clean version)**
	costs (and benefits) beyond the NHS and PSS perspective. As the reviewer mentioned in the following comment that exploration of additional perspectives has beyond what can be drawn from this protocol paper, we feel more comfortable not suggesting the direction of change in the context of this study protocol.	
I agree that a protocol paper is not the place to conduct or fully explore these additional perspectives. However, when discussing limitations, it might be helpful to clarify that the exclusion of these elements (productivity losses and environmental impacts) may influence the interpretation of cost-effectiveness results. If the authors consider this reasoning appropriate, they might also consider slightly refining the relevant sentence in the discussion to reflect that these factors may not affect both arms equally and may warrant more explicit consideration when reporting the full trial results.<br>You may wish to consider rephrasing it along the lines of:<br>"While a societal perspective (including consideration of productivity losses, out-of-pocket expenses, and environmental impacts) is beyond the scope of this evaluation study, these factors may not affect both trial arms equally. Their exclusion should therefore be recognised as a limitation and may warrant more explicit consideration when interpreting and reporting the full trial results."	We agree with the reviewer that it is important to explicitly consider how the interpretation of the cost-effectiveness results can be influenced in those jurisdictions that mandate a broader perspective.<br><br>We also agree that the relevant sentence in the discussion could be improved to reflect that these factors (including consideration of productivity losses, out-of-pocket expenses, and environmental impacts) may not have equal influence on both arms from a societal perspective. We thank the reviewer for this helpful suggestion and have revised the corresponding paragraph in the discussion section.	Discussion<br>Page 12 line 1-6<br>*'From a societal perspective [69], the indirect costs such as out-of-pocket and productivity losses, as well as the environmental impacts of device manufacturing, replacement, and end-of-life disposal, may not affect both treatment strategies equally. While a societal perspective is beyond the scope of this evaluation study, an explicit consideration of these additional costs (and benefits) will be necessary to inform funding decision in those jurisdictions that mandate a broader perspective than the one adopted in the United Kingdom.'*
